# The Application of Microfluidic Technologies in Aptamer Selection

**DOI:** 10.3389/fcell.2021.730035

**Published:** 2021-09-17

**Authors:** Yang Liu, Nijia Wang, Chiu-Wing Chan, Aiping Lu, Yuanyuan Yu, Ge Zhang, Kangning Ren

**Affiliations:** ^1^Department of Chemistry, Hong Kong Baptist University, Kowloon, Hong Kong, SAR China; ^2^Guangdong-Hong Kong Macao Greater Bay Area International Research Platform for Aptamer-Based Translational Medicine and Drug Discovery, Hong Kong, Hong Kong, SAR China; ^3^School of Chinese Medicine, Law Sau Fai Institute for Advancing Translational Medicine in Bone and Joint Diseases, Hong Kong Baptist University, Hong Kong, Hong Kong, SAR China; ^4^Institute of Research and Continuing Education, Hong Kong Baptist University, Shenzhen, China; ^5^State Key Laboratory of Environmental and Biological Analysis, Hong Kong Baptist University, Kowloon, Hong Kong, SAR China

**Keywords:** aptamer, oligonucleotide, SELEX, microfluidics, microarray

## Abstract

Aptamers are sequences of single-strand oligonucleotides (DNA or RNA) with potential binding capability to specific target molecules, which are increasingly used as agents for analysis, diagnosis, and medical treatment. Aptamers are generated by a selection method named systematic evolution of ligands by exponential enrichment (SELEX). Numerous SELEX methods have been developed for aptamer selections. However, the conventional SELEX methods still suffer from high labor intensity, low operation efficiency, and low success rate. Thus, the applications of aptamer with desired properties are limited. With their advantages of low cost, high speed, and upgraded extent of automation, microfluidic technologies have become promising tools for rapid and high throughput aptamer selection. This paper reviews current progresses of such microfluidic systems for aptamer selection. Comparisons of selection performances with discussions on principles, structure, operations, as well as advantages and limitations of various microfluidic-based aptamer selection methods are provided.

## Introduction

An aptamer is a sequence of single-strand oligonucleotide (DNA or RNA) with a variable region of about tens of nucleotide bases. These random sequences endow each aptamer with a unique three dimensional structure and potential binding capability to target molecules, such as metal ions (Rupcich et al., 2006; Noeske et al., 2007; Sun et al., 2017), chemical compounds (Brockstedt et al., 2004; Tang et al., 2006), proteins (membrane protein, circulating protein) (Green et al., 1996; Maberley, 2005), cells (infected cells, stem cells, and cancer cells) (Wiegand et al., 1996; Blank et al., 2001; Wang et al., 2003; Shangguan et al., 2006), and whole micro-organisms (Bruno and Kiel, 1999; Homann and Göringer, 1999; Shangguan et al., 2006).

With their high affinity to specific target, aptamers can block or disrupt the interactions between their targets and other substrates. Hence, in the fields of analysis and diagnostics aptamers can be utilized for molecular recognition against their targets (Keefe et al., 2010). Virous aptamer-based biosensors have been designed for targeted pathogen, drug delivery, cancer diagnosis, and environmental contamination. For instance, fluorophore-doped aptamers were developed to detect enterotoxaemia *E. coli* (ETEC) K88 (Bruno et al., 2010). Similarly, aptamers against cancer biomarkers, such as carcinoembryonic antigen (CEA), MCF-7 breast cancer cells, and prostate specific antigen (PSA) were developed to realize reliable and timely cancer diagnosis (Wang et al., 2015; Yang et al., 2015; Zhang et al., 2015). Consequentially, with their small size, ease of production, thermal stability, minimal restrictions for targets, and the possibility to regenerate, aptamers are considered promising alternatives to the traditional antibodies, and have become increasingly popular in the applications for therapeutics. Pegaptanib, an aptamer specific for vascular endothelial growth factor (VEGF), was licensed in 2000 as Macugen^®^ by OSI Pharmaceuticals for age-related macular degeneration treatment (Ng and Adamis, 2006). Moreover, CH6 aptamer–functionalized lipid nanoparticles (LNPs) were developed as a new RNAi-based bone anabolic strategy, and was granted orphan drug designation by the United States Food and Drug Administration (FDA) as a novel aptamer treatment of osteogenesis imperfecta (OI) (Liang et al., 2015).

**FIGURE 1:**
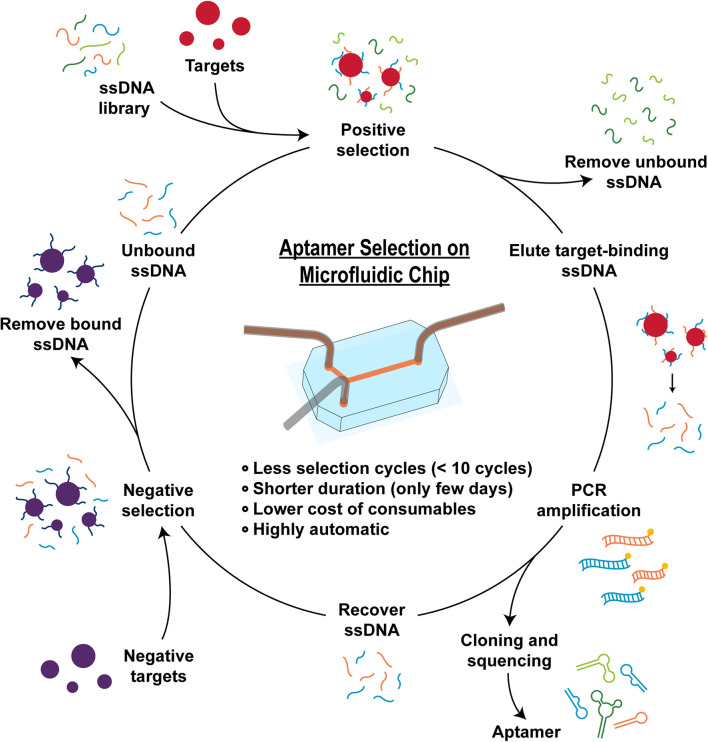
Scheme of aptamer selection mechanism. The aptamer selection (SELEX) processes usually consist of four major steps: preparing a library of randomly generated oligonucleotide sequences, incubating with the target ligand, eluting bound sequences, amplifying the bound oligonucleotides by PCR, and obtaining ssDNA ready to undergo next SELEX cycle. Sometimes, negative selections are added to remove the non-specific binding sequences from the pool. With microfluidic-based SELEX, small amounts of fluids can be precisely manipulated inside the microfluidic devices, which can reduce the cost, improve the speed, increase the resolving power, and upgrade the extent of automation compared to the conventional SELEX processes.

So-called aptamers are often generated by combinatorial chemistry and molecular biology technique called systematic evolution of ligands by exponential enrichment (SELEX). Through using SELEX, tens of aptamer candidates can be selected from a massive library with initially 1014–1015 random sequences. However, although various optimization attempts have been made by the scientists since the debut of SELEX in 1990 (Ellington and Szostak, 1990; Tuerk and Gold, 1990), the conventional SELEX methods still suffer from expensive reagents, high labor intensity, low operation speed/efficiency, and low success rate. In order to overcome these limitations, different innovative approaches have been developed (Stoltenburg et al., 2007). Among these methods, microfluidic-based SELEX has been attracting increasing attention owing to its remarkable advantages. With microfluidic-based SELEX, small amounts of fluids can be precisely manipulated inside the microfluidic devices, which have reduced the cost, improved the speed, increased the resolving power, and upgraded the extent of automation compared to the conventional SELEX system (Figure 1; Lin et al., 2014). On the other hand, microfluidic SELEX methods are still at the early stage of development; further advances are expected to release their potential in large scale implementations.

This review focuses on the current development and application of microfluidic technologies in aptamer selection. We summarize different conventional SELEX and microfluidic-based SELEX approaches; meanwhile, we discuss various microfluidic-based aptamer screening methods in detail, including their principles, designs, operations, as well as their advantages and limitations (Supplementary Table 1). It is noteworthy that great efforts have been made to optimize the design of microfluidic SELEX devices to improve the incubation and partition efficiency. More specifically, the binding and dissociation between the aptamers and the targets is a key part in the incubation and partition process, where microfluidic technologies can play an important role. Various electric fields, magnetic fields, acoustophoresis effects, and hydrodynamic forces have been employed to enhance the efficiency of incubation and partition. Although there are some nice reviews discussing the different types of microfluidic SELEX schemes and applications of aptamer-based microfluidic biosensors (Dembowski and Bowser, 2018; Fraser et al., 2019), there still lacks a review to systematically summarize the microfluidic incubation and partition methodologies and explain their respective pros and cons in different types of SELEX applications. Current review is prepared for this demand, aiming to provide a useful summary for microfluidic experts to introduce the opportunities in aptamer selection, and in the meantime, an understandable guide for SELEX performers to consider microfluidic technologies to upgrade their methodologies. Hereby, we have classified microfluidic aptamer selection schemes into two major types, i.e., microarray chips and automatic driven microfluidic chips based on their differences in screening mechanism and design features. As known, the whole SELEX process should contain several procedures; however, most of the reported microfluidic-based SELEX systems only performed the selection step on chip. In this regard, besides the on-chip selection microfluidic devices, we also discuss in a separate section with several examples of integrated full-SELEX microfluidic systems, which contain the PCR and ssDNA regeneration steps commonly performed off-chip in other reports. Finally, we summarize the current microfluidic-based SELEX applications and discuss the prospect of its future development.

## Overview of Conventional SELEX Technologies

A wide variety of SELEX techniques have been developed to improve the effectiveness and efficiency of the aptamer discovery process. Some of the aptamer selection methods are based on well-known analytical techniques, such as nitrocellulose filter binding and chromatography. Other technologies use magnetic beads and electrophoresis, which usually show improved efficiency compared with the previous methods.

The conventional SELEX processes usually consist of four major steps: preparing a library of randomly generated oligonucleotide sequences, incubating with the target ligand, eluting bound sequence, and amplifying the bound oligonucleotides by PCR. In the incubation step, a single-stranded oligonucleotide library with random nucleic acid sequences (10^12^–10^15^ different sequences) is exposed to the target ligands, which could be small-molecules, proteins, or small organic compounds (Zhang et al., 2009). Aptamer sequences that have bound to the target are collected during the partitioning step, whereas the unbound or weakly bound sequences are removed (Tuerk and Gold, 1990). Bound aptamers are then amplified to prepare to produce an enriched pool and obtain ssDNA ready to undergo next SELEX cycle. Sometimes, negative selections are added to remove the non-specific binding sequences from the pool, e.g., those sequences with affinity to target immobilization matrix components (Figure 1). Typically, if the selection process is successful, after 8–20 cycles (several weeks to months) the enriched aptamer pool is dominated by the strongest binding aptamers, which are identified through sequencing the final pool. These aptamers are then harvested as candidate aptamers and characterized for their binding affinity and specificity. Aptamers with desired properties are then subjected to further optimization for therapeutic and diagnostic applications.

### Nitrocellulose Filter Binding Methods

Most of early studies of SELEX used nitrocellulose filtration to select aptamers (Carey et al., 1983). This technique incubates the target and nucleic acid library together until the solution reaches equilibrium. Afterward, the unbound aptamers are removed from the initial library after the solution passes through some filters (Giver et al., 1993; Conrad et al., 1996). Although this method is simple and does not require any special instrument, it is only suitable for protein targets that non-specifically bind to nitrocellulose, while other target molecules such as small molecules cannot be captured by the filter. In addition, a large number of selection rounds (8–20 rounds) are often needed for the nitrocellulose filter binding method.

### Magnetic Bead-Based Methods

Magnetic separation systems integrated with functionalized magnetic adsorbent particles have also been considered as a useful method in SELEX (Nieuwlandt et al., 1995). The immobilization on magnetic beads makes the SELEX operation easy to hand. By using very small amounts of target molecules and stringent washing condition, rapid and efficient partition of bound and unbound aptamers can be achieved (Wilson et al., 2014). However, there is limitations in incubation that target or aptamer immobilization is restricted with interaction surface.

### Capillary Electrophoresis Methods

In capillary electrophoresis (CE) selections, several nanoliters of the incubation mixture of target molecules and random sequences are injected into a capillary and experience a mobility shift, migrating at different rates, which allows the separation and collection of high-affinity aptamers out of the aptamer library. CE SELEX offers several advantages over conventional selection protocols (Mendonsa and Bowser, 2004; Mosing et al., 2005). This method can isolate high-affinity aptamers in fewer cycles, without cumbersome negative selection, and shorten the process from several weeks to just couple of days. However, the library size is limited to nanoliters to prevent overloading, which can increase the concentration of both target and aptamer molecules and lead to non-specific interactions. What’s more, if the screening time is not strictly controlled, both bound and unbound oligonucleotides will reach the export of CE, which may lead to low efficiency of library selection and enrichment.

### Cell-SELEX Methods

All conventional SELEX methods described above are designed for the aptamers binding to purified protein targets. However, it has been observed that if protein-targeted aptamers are selected through this way, they often fail to recognize their targets in live cells, resulting in failure of the bio-medical application. Therefore, cell-SELEX methods were developed to select aptamers targeting proteins that stay in their natural environment on the cell surface. Moreover, cell-SELEX can also be employed to recognize a particular type of cell and discover novel biomarkers or unknown membrane proteins of cells. The procedure of cell-SELEX is similar to traditional protein SELEX but the operation is more complex (Chen et al., 2016). Cell-SELEX uses whole live cells as targets and therefore appropriate condition for cell manipulation is very essential. After positive selection, Negative selection is commonly adopted in cell-SELEX by removing non-specifically bound oligonucleotide sequences using control cell lines.

## Microfluidic-Based SELEX Technologies

Though the majority of the published aptamers were manually selected, the entire process of obtaining high-affinity and target-specific aptamers is repetitive, time consuming, and not applicable to high-throughput selections. Accordingly, considerable efforts have been made to optimize the selection process, reduce the number of rounds of selection (less than 10 rounds), and successfully obtain desired aptamer sequences within a few days. Microfluidic systems, with the property of small liquid volume, large surface-to-volume ratio, and short diffusion distance, can dramatically accelerate the diffusion process, increase the incubation and separation efficiency while reducing reagents consumption. Furthermore, with multiple functions, such as versatile and accurate liquid manipulation, utilizing interface phenomenon, and possibility of incorporating different physical fields (e.g., electrical, magnetic, thermal, and acoustical), novel mechanisms of aptamer selection could be established. Moreover, selection process has the potential to be miniaturized and streamline by a customized microfluidic device which realizes automated control and multiplexing. Therefore, a variety of microfluidic technologies has been explored to offer improved resolving power to the aptamer selection (Supplementary Table 1).

### Microarray Chip SELEX

Microarray chips are microchip surfaces that are printed with thousands of tiny spots in defined positions, with each spot immobilizing a target. This immobilization can either be based on adsorptive interactions, force interactions or covalent attachment. The targets attached to each area of a chip surface are usually protein or small molecules for either positive or negative selection to bind ssDNA library. No complicated structure design and operations were needed in microarray chip SELEX.

#### Protein Microarray-Based Selection

**FIGURE 2:**
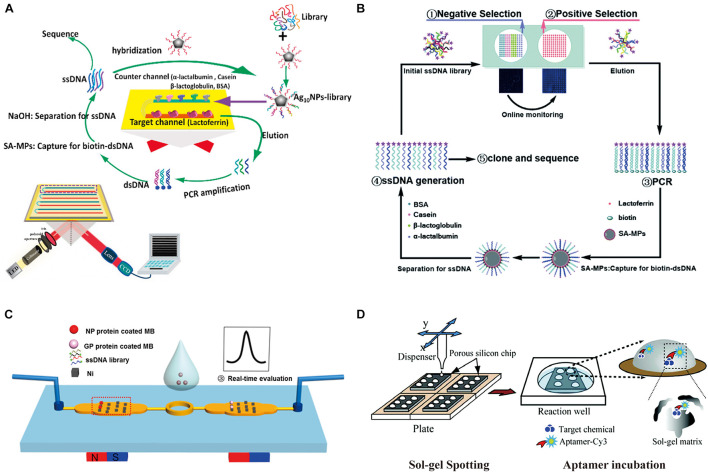
Microarray chips for aptamer selection. **(A)** Schematic diagram of the Ag10-NPs-library SPRI-microfluidic-SELEX method. The biochip consists of eight parallel channels on an SAM-gold substrate sealed in a customized flow cell. **(B)** Principle of microarray-SELEX for aptamer screening. **(C)** Magnetism-array selection chip. Reprinted with permission from Hong et al. (2017). Copyright 2018 American Chemical Society. **(D)** Schematic of the PS–SG *in vitro* selection aptamer chip, where small target molecules are entrapped in a spotted sol–gel microarray, which is then incubated with aptamers.

Danke Xu’s group developed a protein microarray microfluidic chip for screening aptamers, which is called PMM-SELEX (Liu et al., 2017). Positive target protein and negative proteins were dotted and immobilized by physical adsorption separately to form the protein microarray. This prepared microarray was then integrated into a microfluidic chip to screen the potential aptamers for target protein lactoferrin. The interaction between aptamer candidates and the targets were monitored by fluorescent scanner. Later the same group reported a novel approach (Figure 2A) that combines Ag10-NPs enrichment and SPR imaging (SPRI) with the SELEX process to evaluate the detection signal (Jia et al., 2018). Subsequently, to solve the problems of complicated manufacturing and instrumentation, this group established a novel microarray SELEX platform (Yu et al., 2019) with a Smart Arrayer and Microarray Scanner to simplify fabrication and experimental operation (Figure 2B).

#### Magnetism-Controlled Microarray-Based Selection

In addition to directly immobilizing the protein targets onto a chip surface, Zhi-Ling Zhang’s group took advantage of magnetic nanospheres (MNs) patterned chips to develop a screening platform with multiple functions (Hong et al., 2017). The MNs were manipulated in the micrometer scale to bind with mucin 1 (MUC1) as the target protein, which showed fast binding, low non-specific adsorption, rapid magnetic response, and the ability to undergo continuous stringency washing to effectively remove weakly bound aptamers (Figure 2C). Specific aptamers for MUC1 with the *K*_*d*_ of 22 nM were obtained through only two rounds of selection. To more precisely control the parameters that affect the efficiency of selection, the same group invented magnetism-controlled selection chips which generated separation efficiency about a 10-fold improvement over the previous method (Hong et al., 2019).

#### Sol–Gel Microarray-Based Selection

Sol–gel technology has also been incorporated into microfluidic devices to provide a matrix for target immobilization. Sol–gel is a silicate material formed by the chemical reaction of hydrolysis and polycondensation that contain both nanoscale pores and micro-pore channels (Chen et al., 2016). In SELEX, aptamers can freely move through the micro-pore channels in sol–gel, but are retained inside the sol–gel through interaction with targets entrapped in nanoscale pores, which provide an environment for keeping both aptamers and targets in their native states while promoting their intermolecular interaction.

The first sol–gel array microfluidic system for SELEX imbedded various target proteins into nano-porous sol–gel droplets to integrate microarrays on microfluidic chip. The captured protein–aptamer complexes were then selectively eluted with the help of electrical micro-heaters at the bottom of the sol–gel chip (Park et al., 2009). This design of sol–gel array microfluidic system improved the selection efficiency, producing high-affinity aptamers of TATA-binding protein (TBP) by 5–8 selection rounds (Ahn et al., 2011). Later, a sol–gel chip combined with a new porous silicon substrate (PS–SG chip) was applied to provide a strong anchoring between the sol–gel matrix and the substrate, and prevent the problem of low adhesiveness of the sol–gel matrix to conventional substrates (Figure 2D). Using this PS–SG chip, an aptamer for targeting BPA was successful acquired by binding inside the sol–gel matrix (Ahn et al., 2012) and specific aptamers against xanthine with sensitivity of detection as low as 1 mM were isolated (Bae et al., 2013). Besides substrate adhesion, cross-contamination was another problem for sol–gel microfluidic SELEX. To prevent these problems, a microfluidic network platform equipped with pneumatic valves was developed to allow the serial loading and incubation of aptamers. At the eluting step, the cross-contamination-free parallel elution was performed by sealing the pneumatic microvalves between adjacent elution chambers. Specific aptamer binding to human HSF1, Yeast TBP, and enhanced GFP were eluted in parallel without any cross-contamination (Lee et al., 2013).

Despite continuous progresses, there is still room to improve. Among the majority of the works based on microarray-SELEX, the interaction and partition mechanism between protein and DNA is in a relatively static and loose manner, leading to a long incubation time and low selection efficiency. Besides, the chips’ structure and use were generally complicated.

### Automatic Force Driven Microfluidic SELEX

Most efforts to upgrade the SELEX strategies have been applied to improve the separation efficiency between the target-bound single strand nucleic acid and unspecific nucleic acid molecules. A better incubation and separation efficiency can lower the non-specific binding and accelerate the enrichment of the aptamer candidates in each selection cycle. Applying contactless external driving forces and hydrodynamic forces to microfluidic systems can provide better ways to enhance the efficiency of aptamer selection through tailored fluid manipulation, incubation, and partition process control. Thus, various novel microfluidic SELEX techniques have been developed, which take advantage of hydrodynamic and other force fields such as electric, magnetic, and acoustic fields, and improved the capability in generating high-performance aptamers.

#### Force Field Driven Microfluidic Selection

**FIGURE 3:**
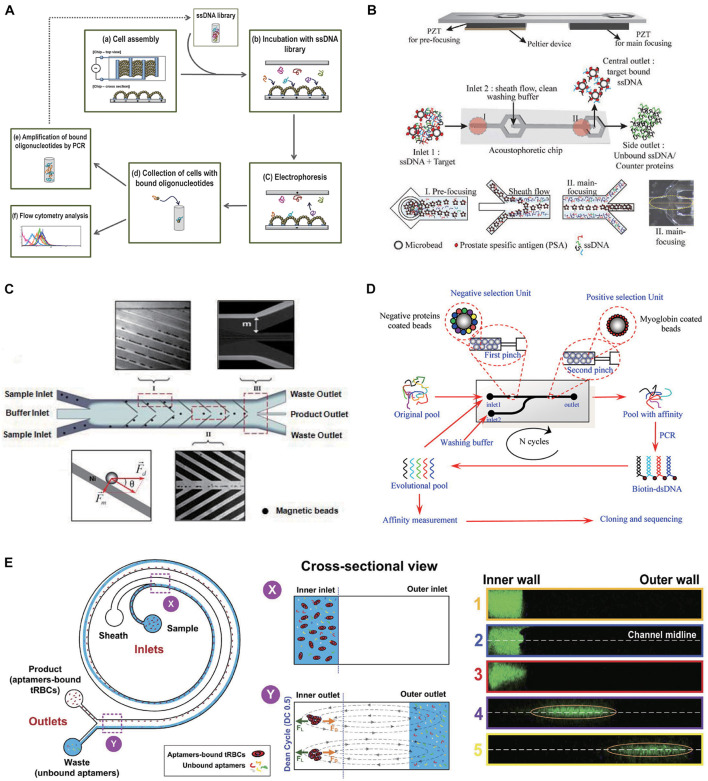
Force field driven microfluidic chips. **(A)** Dielectrophoresis and electrophoresis combined microfluidic chip for performing cell-SELEX procedure. **(B)** The schematic overview of the acoustophoretic device and SELEX process. **(C)** Design of the CMACS microfluidic SELEX device. **(D)** Myoglobin-aptamer selection microfluidic chip. The positive and negative selection unites are integrated in one micro-channel. Reprinted with permission from Wang et al. (2014). Copyright 2014 American Chemical Society. **(E)** Inertial microfluidic SELEX (I-SELEX) utilizing Dean vortices to select aptamers for red blood cells (RBCs).

In the incubation process of SELEX, microfluidic systems incorporated with contactless external driving forces can mix all the fluidic samples rapidly in an automated manner. In the partition process, strong force generated by microfluidic systems can pull away weakly bound oligonucleotides from the target and manipulate fluid precisely to achieve high selection efficiency. Methods of enhancing incubation with electric, magnetic fields, and field gradients have been increasing over the years. Avci-Adali’s group designed a microfluidic device combining dielectrophoresis and electrophoresis to perform cell-SELEX procedure (Stoll et al., 2015). Dielectrophoresis controlled by the electrodes can drive the target cells to selectively assemble to the proper chamber for incubation with aptamers (Figure 3A). On the other hand, the electrophoretic field can remove the loosely bond aptamers with low affinity. In addition to electrophoresis, acoustophoreis has also been applied for aptamer selection. Thomas Laurell’s group developed a novel microfluidic chip for screening aptamers for a prostate-specific antigen based on an acoustofluidic separation method (Park et al., 2016). Acoustophoresis enables a standing wave field to trigger particle movement. It creates a rapid and continuous flow-based process for the simultaneous partition and washing, and thus an efficient flow-based process for the aptamer isolation. As the beads-protein-ssDNA compounds entered the acoustic standing wave field, the micro-particles with target bound DNA-fragments migrated across the central buffer interface and exited the system through the central outlet led by the acoustic radiation force, whereas the unbound proteins and ssDNA remained in the original buffer stream along the side walls and were removed through the side outlets (Figure 3B). PSA binding aptamer was obtained with a *K*_*d*_ of 0.7 nM to PSA. Despite force field driven microfluidic selection improves the efficiency in tailored fluid manipulation, incubation, and partition process control, sophisticated apparatuses are needed to simulate the force control, which increase the complexity of the SELEX system. Also, one restriction is that force fields sometimes result in a standing wave field that extends along the whole fluid channel. Thus, it is difficult to adjust the intensity and distribution of force field to precisely guide the moving paths of particle (Tsutsui and Ho, 2009). Furthermore, force field manipulation within a microfluidic channel could result in temperature increase which may bring adverse effects on molecule and selection results (Evander et al., 2007).

#### Hydrodynamic Driven Microfluidic Selection

Recently, the hydrodynamic-based microfluidic SELEX chips are of increasing attention. In this technique, hydrodynamic forces are generated based on the design of channel structure. For SELEX, target-loaded beads for exposure to aptamer library can be trapped in a particular position of the chip and separated by pre-designed fluidic flow without the application of any external force field such as electric, magnetic, or acoustic. H. Tom Soh developed a microfluidic system to perform magnetic bead-assisted SELEX. Aptamers with high-affinity can be obtained through only a single round of selection (Lou et al., 2009). Some ferromagnetic microstructures were patterned on the device. In the separation step, magnet field gradients enable magnetophoretic forces and hydrodynamic forces properly balanced to accurately and reproducibly manipulate very small amount of magnetic particles and target protein with high purity (high ratio between the amount of DNA collected from the Waste outlet compared with those collected from the product outlet), recovery rate, and throughput. The device takes advantage of multi-stream laminar fluidic architecture to yield extremely high molecular separation efficiencies (Figure 3C). This group further developed a micromagnetic separation (MMS) chip (Qian et al., 2009) to avoid the problems of microbubbles distorting the flow streams and bead aggregations blocking the microchannel, which could influence the purity and recovery of aptamers. Using this modified MMS chip, aptamers have been selected that specifically bind platelet (Ahmad et al., 2011) derived growth factor B (Cho et al., 2010), thrombin, apolipoprotein E3 (ApoE), and streptavidin (Oh et al., 2011) with *K*_*d*_ of 0.028, 0.33, 3.1, and 35.2 nM.

The mentioned selection mechanism of MMS chip is not only driven by hydrodynamic force, but also manipulated using magnet field. There are also some works performed SELEX solely using hydrodynamic-based microfluidic chips with specially designed structure and uncomplicated instruments. Wang et al. constructed a microfluidic platform that integrate the positive and negative selection units in one channel (Wang et al., 2014). Beads coated with positive and negative selection proteins were blocked in front of two pinches, respectively, while the ssDNA library was injected into the channel and eluted from the outlet to achieve positive selection and negative selection simultaneously (Figure 3D). DNA aptamers for Myoglobin were successfully isolated after seven circles of selection. The highest affinity of the aptamers selected by this platform reached the *K*_*d*_ of 4.93 nM. Birch et al. (2015) demonstrated an inertial microfluidic SELEX (I-SELEX) based on inertial focusing realized in microfluidic channels with a bi-loop spiral. This channel design can achieve effective partitioning of red blood cells (RBCs) from unbound aptamers at a high volume throughput (Birch et al., 2015). In the bi-loop channel, unbound aptamers move along with Dean vortices toward the outer wall and to the waste outlet, whereas the target beads/cells with bound aptamers are lifted toward the inner wall and to the product outlet (Figure 3E).

Although these hydrodynamic driven microfluidic systems for aptamer selection had low power consumption and streamlined system design, most of them were based on the inertial microfluidics and Dean-flow schemes, which showed limited parallelization power and selection throughput. Therefore, facile and versatile microfluidic systems that provide sufficient selection throughput and versatile operability with are still demanded.

### Integrated Full-SELEX Microfluidic Systems

Although the microfluidic systems discussed above improved the sensitivity and specificity of SELEX compared to the traditional methods, they only performed the library extraction process on chip. Off-chip procedures require extra time and labor consuming, which obstruct the increase of the overall SELEX efficiency. In this regard, integrated microfluidic systems with the ability to perform the entire iterative screening process of SELEX are still in great demand. However, fully integrated microfluidic chips for SELEX require automated synergy of system components, such as valves, pumps, heaters, mixers, and sensors, which increases the complexity of such microfluidic systems to be fabricated. Some groups have successfully designed workable, self-contained, microfluidic chip-based systems capable of carrying out automated SELEX process.

**FIGURE 4:**
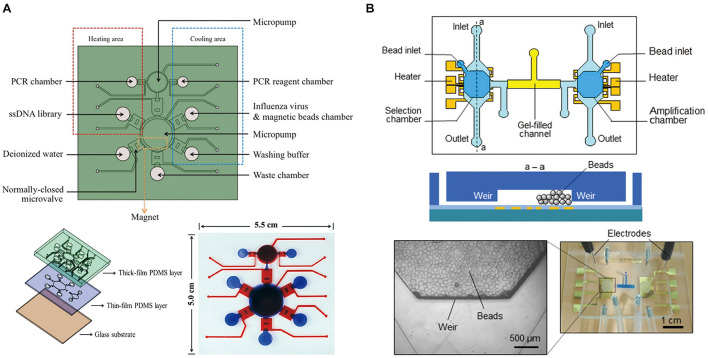
Integrated full-SELEX microfluidic systems. **(A)** Schematic diagram of the integrated microfluidic SELEX chip. It consisted of two PDMS layers and one glass substrate. **(B)** Integrated microfluidic SELEX. It includes a selection microchamber and an amplification microchamber interconnected by a gel-filled microchannel: top and cross-sectional schematics. A micrograph of the microchip with microbeads retained in the microchambers and agarose gel (dyed blue for visualization) filling the interconnection channel. Inset: Micrograph of the weir-like flow constriction region with retained beads.

Hybarger et al. (2006) first reported a microfluidic SELEX prototype comprised of an on-chip nucleic acid amplification device and a robotic manipulator to deliver reagents to each part of workstations, where the individual steps of the SELEX procedures are performed to carry out automated SELEX. This microfluidic SELEX prototype has paved the way for subsequent automated microfluidic systems for full process SELEX with optimized accuracy control and miniaturization. Huang et al. (2010) presented an automatic, microfluidic platform based on magnetic beads, which integrates a random ssDNA extraction device and a micro-PCR machine for fast screening aptamers with specific binding to C-reactive protein (CRP). It is a three-layer, four-chamber PDMS and glass chip integrated with three major modules. Later, they reported a new active micromixer actuated by compressed air, which can deflect the PDMS membrane chamber and was adopted to incubate magnetic beads with ssDNA (Huang et al., 2012). A circular micropump featuring a normally closed microvalve was applied for precise and rapid fluid moving and mixing. Microheaters and an integrated micro-temperature sensor were incorporated to control the temperature for PCR process. This modified system enhanced the aptamer amplification yield and successfully selected the AFP-specific aptamers with the dissociation constant of 2.37 nM. Weng et al. (2013) applied this system to select cell aptamers. To keep the target cells alive during the cell-SELEX process, suction-type pneumatic micro-chambers for gentle and efficient incubation were applied; the *K*_*d*_ of the obtained specific aptamer for cancer stem-like cells was 15.32 Nm (Figure 4A). In addition to protein and cancer targets, aptamer probes against influenza A virus (Lai et al., 2014), cardiovascular biomarkers (Sinha et al., 2018), and ovarian cancer tissues (Liu et al., 2019) have also been automatically selected with this integrated microfluidic system. The microfluidic system for automated SELEX against ovarian cancer tissue integrates six micromixers onto one single chip to perform six selection rounds in a continuous fashion. This design streamlines the selection process, and avoids the complicated calibration before each run. Three aptamers against ovarian cancer tissues were successfully screened with the *K*_*d*_ 1348.0, 129.2, and 178.0 nM, respectively. Compared to above mentioned microfluidic systems which contain one or two operation steps of SELEX, the integrated microfluidic SELEX systems have improved the automation and accelerated the aptamer synergy of system components, such as valves, pumps, heaters, mixers, and sensors, which increases the complexity of such microfluidic systems to be fabricated. Efforts to develop streamlined and miniaturized full-SELEX microfluidic system are still in need.

Reported integrated microfluidic systems could perform most parts of the SELEX process, but the regeneration of single-stranded nucleic acid from PCR products between two sequential rounds of SELEX have usually been missing. Lin’s group developed an integrated microfluidic system involving the ssDNA regeneration process, which employed a microbead-based method to select and amplify DNA aptamers. It uses an electrophoretic DNA manipulation scheme to couple the entire SELEX process (Kim et al., 2016). The microfluidic chip contains heaters and temperature sensors integrated onto selection and amplification chambers, and some parts of the channel is filled with sol–gel (Figure 4B). Strong binders to the target are transferred between selection and amplification chambers by electrophoretical force through the gel-filled microchannel while microbeads are retained inside the microchambers. The isolated aptamer candidates show strong target-binding affinity, with *K*_*d*_ of 12 nM to immunoglobulin E (Olsen et al., 2017).

## Conclusion

The application of microfluidic technologies offers the advantages of high speed, low cost, and labor-saving, which can contribute strongly to the development of aptamer selection; with the utilization of hydrodynamic, electric, magnetic, and acoustic force fields, they can also provide superior performance compared with traditional large-scale laboratory apparatus. There have been some nice progresses in the techniques for microfluidic incubation, separation, and amplification, which showed encouraging promise to improve the efficiency of SELEX. Current trend in microfluidic SELEX design is to expand from a chip for the most critical partition step only to an integrated full-SELEX system for performing successive selection rounds and avoiding errors caused by manual operation. Advances in the development of easy-to-use and automated SELEX procedures will encourage researchers to select applicable aptamers for more targets.

Despite great efforts in developing these novel strategies, microfluidic SELEX technologies in aptamer selection are still not widely harnessed in general biological laboratories or clinical applications. One major reason is the commonly low success rate of obtaining the desired aptamers. Currently, there still lacks an effective method to overcome this problem, but the loss of potential aptamer sample during the SELEX process through fouling the chip material might be one important reason of the failure. Addressing this problem could be helpful to increase the success rate of the selection. On the other hand, it is apparent that systematic development is still needed to establish fully integrated and reliably functional automated microfluidic SELEX systems. Recently, a number of disease-biomarker aptamers were successfully selected by microfluidic technology, such as platelet derived growth factor BB (PDGF-BB) protein for various pathological states, myoglobin for cardiovascular disease, and alpha-fetoprotein for liver cancers (Huang et al., 2010, 2012; Weng et al., 2013). Besides, aptamers as therapeutics, such as aptamer specific to MUC1 for anticancer therapy were also isolated using microfluidic technology (Hong et al., 2019). With more SELEX platforms based on novel microfluidic technology to be developed in the near future, we envision practical and even commercialized implementation of automated SELEX technology to promote aptamer-based biosensing, biotechnology, and medicine.

## Author Contributions

GZ, KR, and YY conceived the idea. YL, NW, and KR wrote the initial draft. All authors reviewed, commented, and revised the manuscript.

## Conflict of Interest

The authors declare that the research was conducted in the absence of any commercial or financial relationships that could be construed as a potential conflict of interest.

## Publisher’s Note

All claims expressed in this article are solely those of the authors and do not necessarily represent those of their affiliated organizations, or those of the publisher, the editors and the reviewers. Any product that may be evaluated in this article, or claim that may be made by its manufacturer, is not guaranteed or endorsed by the publisher.
