# Supplementary material for: The Application of Microfluidic Technologies in Aptamer Selection
Source: Front Cell Dev Biol. 2021 Sep 17;9:730035. doi: 10.3389/fcell.2021.730035 (PMC8484746; doi:10.3389/fcell.2021.730035)
Supplement: Supplementary file 1 [file Data_Sheet_1.PDF]

Supplementary Material

The application of microfluidic technologies in aptamer selection

Yang Liu [1,3], Nijia Wang [1,3], Chiu-Wing Chan [1], Aiping Lu [2,3], Yuanyuan Yu [2,3]*, Ge Zhang [2,3]*, Kangning Ren [1,3, 4,5]*

[1]Department of Chemistry, Hong Kong Baptist University, Waterloo Rd, Kowloon, Hong Kong, China.

[2]Law Sau Fai Institute for Advancing Translational Medicine in Bone & Joint Diseases, School of Chinese, Hong Kong Baptist University, Hong Kong SAR, China

[3]Guangdong-Hong Kong Macao Greater Bay Area International Research Platform for Aptamer-based Translational Medicine and Drug Discovery, Hong Kong SAR, China

[4]HKBU Institute of Research and Continuing Education, Shenzhen, China
[5]State Key Laboratory of Environmental and Biological Analysis, The Hong Kong Baptist University, Waterloo Rd, Kow-loon, Hong Kong, China

*** Correspondence:**
Corresponding Authors
yuyuanyuan@hkbu.edu.hk (YY); zhangge@hkbu.edu.hk (GZ); kangningren@gmail.com (KR)

Supplementary Table 1. Brief description and comparison of microfluidic SELEX methods

Microfluidic SELEX Method	Aptamer	Target	Character	Advantage(s)	Disadvantage(s)	Reference
Protein microarray-based Microfluidic SELEX	DNA	Protein (Lactoferrin)	●A small volume of biological macromolecules of each spot in the array are printed on a carrier to serve as the target.	● Sufficient affinity and amount of targets (Thousands of microscopic spots could be printed on a chip)<br>●Multiple target screening is performed by immobilization different targets on chip (Some of proteins were used as target protein, while the others proteins were used as negative proteins on the same chip)	● Limited capacity for protein and chip bound (Proteins were dotted on a chip by physical adsorption)<br>● A specific instrument to monitor binding efficiency are required (Laser confocal scanner or a charge-coupled camera)	(Jia et al., 2018; X. Liu et al., 2017; Yu et al., 2019)
Magnetism-controlled microarray-based Microfluidic SELEX	DNA	Protein (MUC1)	●Magnetic nickel patterns are fabricated on the chip surface to control the interaction between beads and target	● Nonspecific binding aptamers are suppressed (The selection incubating process was conducted in a flowing stream)<br>●Applicable to most targets (There are many types of magnetic beads available, which can be used for different proteins)	●Delicate to map the magnetic field and force distribution on the micrometer scale manipulation of micron-sized magnetic beads is needed (Controlling of magnetic field on the micrometer scale must in high-precision)<br>● Small-scale mixing is needed (The liquid mixture could improve the reaction efficiency)	(Hong et al., 2017, 2019)
Sol-gel microarray-based selection	RNA<br>DNA<br>DNA<br>RNA	Protein (TBP)<br>Chemical (BPA)<br>Chemical (Xanthine)<br>Protein (BSA, TBP, TFIIB, HSF1, eGFP)	● of sol-gel. Sol−gels are used for small molecule encapsulation with the goal to conveniently isolate high affinity aptamers against low molecular chemicals. Aptamers are retained by their interaction with targets entrapped within nanoscale pores of sol-gels.	●Target specific aptamers for a wide range of small molecules are potential to developed (Provides optimal retention of targets of various sizes, optimal access of probes)<br>●A variety of biomolecules could be encapsulated (Enzymes, antibodies, regulatory proteins, membrane-bound receptors, nucleic acid aptamers, and even whole cells)<br>●The step of target immobilization is eliminated (Biomolecules are entrapped in the growing covalent silica gel network rather than being chemically attached to an inorganic material)	● Long material gelation times is needed (>2.5H)<br>●Room temperature and humidity are needed to be controlled (To prevent irregular shape deposition due to small deposition volumes and evaporation)<br>● Relatively fragile of the sol-gel material (Limited by brittleness and low porosity)<br>● Not reusable (Sol-gel is a disposable material)	(Ahn et al., 2011; S. M. Park et al., 2009)<br>(Ahn et al., 2012)<br>(Bae et al., 2013)<br>(Lee et al., 2013)
Force field drive microfluidic SELEX	DNA<br>DNA	Cell (HEK-293 cells)<br>Protein (PSA)	● External driving forces (electrophoretic, acoustophoresis) are applied for tailored fluid manipulation, incubation, and partition process control	●The washing steps is simplified (Simultaneous separation and washing)<br>●The nonspecifically bound oligonucleotides are efficient eliminated (Remove of weak binding and	●External energy source and instruments are needed (Need piezoelectric transducing plates and interdigitating array of electrodes)	(Stoll et al., 2015)<br>(J. W. Park et al., 2016)

				nonspecific binding ssDNA)		
Hydrodynamic drive microfluidic SELEX	DNA<br>DNA<br>DNA<br>DNA<br><br>DNA<br>DNA	Protein (BoNT A-rLc)<br>Protein (Streptavidin)<br>Protein (PDGF-BB)<br>Protein (Thrombin, Apolipoprotein E3)<br>Protein (Myoglobin)<br>Cell (RBC)	●Hydrodynamic forces are used to precisely and reproducibly manipulate target and aptamers in flows by taking advantage of the structure and micro dimension of microfluidic chips. A rapid and continuous flow-based process is applied for the simultaneous separation and washing.	●The hydrodynamic is accurately controlled with minimal loss (Optimize the operating flow rates to maximizing recovery)<br>● Accommodate particles of different sizes in microfluidic channels (The device has sufficient tolerance to accommodate a range of particle sizes)<br>●Rapid partitioning of target-bound and unbound aptamers (Product outlet and waste outlet)<br>● Higher affinities for the targets because of stringency conditions (High-shear gradients and secondary Dean flows near the channel wall are enhanced removal of weakly-bound aptamers)	●Complicated design of chip structures (Chip design relates to channel dimensions, curvature, and flow rate)<br>● Scrupulous tuning of the device with microscopy are needed to achieve the high partition efficiency and recovery of bead-bound oligonucleotides (Monitor focusing of the fluorescently labeled aptamer stream by microscopy and control over the free aptamer stream occurs)<br>● Microbubbles in the flow streams and blockage in the micro-channel (Streams and blockage are influence flow rates and bond efficiency)	(Lou et al., 2009)<br>(Qian et al., 2009)<br>(Cho et al., 2010)<br>(Oh et al., 2011)<br><br>(Wang et al., 2014)<br>(Birch et al., 2015)
Integrated full-SELEX microfluidic SELEX	DNA<br>DNA<br>DNA<br>DNA<br>DNA<br>DNA<br><br>DNA	Protein (CRP)<br>Protein (AFP)<br>Virus (InfA,H1N1)<br>Cell (A549)<br>Protein (Immunoglobulin E)<br>Protein (Cardiovascular biomarkers)<br>Tissue (Ovarian cancer)	● The entire SELEX process containing a random ssDNA extraction device and an on-chip nucleic acid amplification is integrated into a microfluidic device for fast screening of aptamers. The device is equipped with micropumps, microvalves and microheaters to control and manipulate the whole SELEX process	●The time-consuming repetitive cycles of SELEX are automated (The microchip could be further instrumented and programmed to allow fully automated operation)<br>●Enable highly efficient isolation of aptamers in drastically reduced times and with minimized consumption of biological material (The entire iterative SELEX process is hence integrated on a single chip and does not require any offline procedures)<br>●Enable a large scale, robust, reproducible and parallelized production of novel aptamers (Selection of aptamers with respect to targets that are either surface-immobilized and solution-borne)	● Sophisticated design and numerous apparatus are needed (Heater, syringe pump and temperature sensors)<br>●High and laborious fabrication cost of the whole system (The whole system includes control system, selection chamber and amplification chamber)<br>● Cross-contamination risk in automated operation (Incomplete washing will affect the next round of screening in chip)	(Chao June Huang et al., 2010)<br>(Chao Jyun Huang et al., 2012)<br>(Lai et al., 2014)<br>(Weng et al., 2013)<br>(Kim et al., 2016; Olsen et al., 2017)<br>(Sinha et al., 2018)<br><br>(W. T. Liu et al., 2019)
